# Elastin calcification during the initial stage of vascular calcification in *kl/kl* mice is suppressed by the calcimimetic upacicalcet

**DOI:** 10.1038/s41598-026-56029-z

**Published:** 2026-06-18

**Authors:** Tomoka Hasegawa, Tomomaya Yamamoto, Xuanyu Liu, Mai Haraguchi-Kitakamae, Hiromi Hongo, Seigo Akari, Takashi Nakamura, Norio Amizuka

**Affiliations:** 1 Ultrastructure of Hard Tissue, Faculty of Dental Medicine, Hokkaido University, Sapporo, Japan; 2 Department of Dentistry, Ground Self- Defense Force, Camp Shinmachi, Takasaki, Japan; 3 Department of Medical Affairs, Sanwa Kagaku Kenkyusho Co., Ltd, Nagoya, Japan

**Keywords:** Vascular calcification, Elastin lamina, *kl/kl* mice, Upacicalcet, Transdifferentiation, Biochemistry, Cell biology, Diseases, Physiology

## Abstract

We performed ultrastructural analyses of the aortae of *klotho*-deficient (*kl/kl*) mice to characterize the early stages of medial calcification and to evaluate the inhibitory effects of the calcimimetic upacicalcet. In the aorta of *kl/kl* mice, calcified materials were localized to the superficial layers of fragmented elastic laminae and were observed as minute, nodule-like elastin aggregates containing calcium phosphate deposits. Although the gene expression of elastin-degrading enzymes and osteogenic markers was increased in *kl/kl* mice, these elastin aggregates were ultrastructurally distinct from conventional matrix vesicles or osteoblast-derived calcifying nodules observed in bone. In contrast, highly calcified regions of the *kl/kl* aorta contained bone-like tissue composed of cells exhibiting osteoblastic, osteocytic, and osteoclastic phenotypes. Administration of upacicalcet attenuated elastin fragmentation and significantly reduced medial calcification in the aorta. In addition to lowering serum calcium and parathyroid hormone levels, upacicalcet reduced serum phosphate concentrations and suppressed the expression of elastin-degrading enzymes and osteogenic markers. Collectively, these findings demonstrate that vascular calcification in *kl/kl* mice involves a complex pathological process comprising passive calcific deposition on degraded elastin and biologically regulated calcification and ossification mediated by osteoblast-like cells and that upacicalcet mitigates disease progression by preserving the structural integrity of aortic elastic laminae.

**Supplementary Information:**

The online version contains supplementary material available at https://doi.org/10.1038/s41598-026-56029-z.

## Introduction

Vascular calcification is a major complication of chronic kidney disease–mineral and bone disorder (CKD-MBD), diabetes, and aging and is strongly associated with adverse cardiovascular outcomes. It is broadly classified into intimal and medial forms, with medial calcification being particularly prevalent in CKD-MBD^1^. Medial vascular calcification, also known as Mönckeberg’s sclerosis, leads to arterial stiffening, impaired vasoreactivity, and increased cardiovascular morbidity and mortality^2,3^. Despite its clinical significance, the mechanisms that initiate medial calcification remain incompletely understood. Clarifying these earliest events is therefore a critical unmet need with therapeutic implications.

Current paradigms suggest that medial calcification is an actively regulated process driven by osteoblastic transdifferentiation of vascular smooth muscle cells (VSMCs) and osteogenic differentiation of undifferentiated mesenchymal cells^4^, accompanied by matrix vesicle-mediated calcification^5,6^. Elevated serum calcium (Ca) and inorganic phosphate (Pi) are considered central drivers of this process. In particular, Pi uptake through type III sodium-dependent phosphate transporters, PiT1 and PiT2, activates ERK1/2 signaling in VSMCs, thereby promoting osteogenic differentiation^7^.

Beyond these signaling events, Pi supply and transport associated with matrix vesicles are likely to play pivotal roles in mineral nucleation. Several enzymes involved in extracellular pyrophosphate (PPi) metabolism contribute to this process^8,9^. In bone, ectonucleotide pyrophosphatase/phosphodiesterase 1 (ENPP1) generates PPi, a potent inhibitor of calcification, from extracellular ATP^10^. PPi is subsequently hydrolyzed by osteoblast-derived alkaline phosphatase (ALP) to produce Pi, thereby increasing local Pi concentrations near matrix vesicles^11–13^. Pi is transported into matrix vesicles through PiT1^9^. In parallel with the extracellular regulation of PPi/Pi balance by ENPP1 and ALP, phosphoethanolamine/phosphocholine phosphatase 1 (PHOSPHO1), which is localized within matrix vesicles, contributes to intravesicular Pi generation^14–16^. However, whether and how these phosphate-regulating systems are spatially and temporally coordinated during the earliest stages of medial vascular calcification remains unclear.

In addition to cellular mechanisms governing Pi regulation, extracellular matrix components, particularly elastic fibers, may act as critical nucleation sites. Under conditions of elevated Ca and Pi, elastin becomes susceptible to degeneration and provides a scaffold for mineral deposition. Elastin-derived peptides promote Ca/Pi deposition and VSMC calcification^17,18^. Consistent with this, Ca binding to elastin has been demonstrated biochemically^19^ and has been linked to physicochemical determinants such as carboxylate density and particle size^20^. Collectively, these findings suggest that structural alterations in elastic fibers may precede, and potentially, facilitate osteogenic differentiation during medial calcification.

The *klotho*-deficient (*kl/kl*) mouse is a unique model for investigating the early events in medial calcification. This model exhibits reduced klotho expression owing to a genetic alteration, leading to impaired fibroblast growth factor 23 (FGF23) signaling, disrupted renal Pi reabsorption, persistent hyperphosphatemia, and elevated serum Ca levels^21^. Notably, *kl/kl* mice develop spontaneous and widespread medial calcification from approximately 6–7 weeks of age, even in the absence of secondary hyperparathyroidism, with progressive worsening over time^21,22^. Reduced klotho expression is a key determinant of CKD-MBD progression^23^, supporting the relevance of this model. Mechanistically, elevated serum Pi is thought to drive calcification through ERK activation, while additional pathways, including BMP2/4 signaling^24^, LRP5/6-mediated Wnt–β-catenin signaling, and FGF-related pathways, collectively promote osteogenic differentiation^25–27^.

Although osteogenic markers and calcification-associated genes are well documented in the vasculature of *kl/kl* mice^24,28^, the ultrastructural and molecular events that initiate medial calcification remain poorly defined. This gap is important because early-stage events are likely to shape disease progression and may represent key therapeutic targets. From a therapeutic standpoint, interventions that can prevent or delay vascular calcification are urgently needed. Calcimimetics have shown anti-calcific effects in experimental CKD-MBD models^29–31^; however, their underlying mechanisms remain incompletely understood.

Based on these considerations, we hypothesized that medial vascular calcification in *kl/kl* mice is initiated by coordinated ultrastructural alterations in elastic fibers and matrix vesicle-associated Pi metabolism and that these early events are modulated by calcimimetic therapy. To test this hypothesis, we aimed to (i) delineate the ultrastructural abnormalities that arise during the earliest phases of medial vascular calcification in *kl/kl* mice; (ii) determine whether the calcimimetic upacicalcet attenuates vascular calcification in this model; and (iii) clarify the ultrastructural mechanisms underlying its anti-calcific effects.

## Materials and methods

### Animals and sample collection

Four-week-old male wild-type (*WT*) and *kl/kl* mice (*n* = 48 total; 24 per genotype; CLEA Japan, Tokyo, Japan) were used in this study. All procedures followed the principles for the care and use of laboratory animals established by Hokkaido University (Approval Nos. 21 − 0013 and 25–0056). This study is reported in accordance with ARRIVE guidelines 2.0, and all experiments were performed in compliance with relevant regulations.

*WT* and *kl/kl* mice were group-housed (3–4 animals per cage) and randomly assigned to receive either vehicle (saline) or upacicalcet—a selective calcium-sensing receptor (CaSR), agonist used to treat secondary hyperparathyroidism in patients undergoing dialysis^32^—while being fed a standard diet containing 1.17% Ca and 0.69% Pi for 3 weeks. Upacicalcet was administered by subcutaneous injection at 1.0 mg/kg/day for 3 weeks, as previously described^31^. This design resulted in four experimental groups (*n* = 12 per group): vehicle-fed *WT* mice (*WT* mice), upacicalcet-fed *WT* mice (*WT*-Upa mice), vehicle-fed *kl/kl* mice (*kl/kl* mice), and upacicalcet-fed *kl/kl* mice (*kl/kl*-Upa mice). All groups were matched for body weight.

Before tissue fixation, mice were anesthetized by intraperitoneal injection of a cocktail containing medetomidine (0.3 mg/kg), midazolam (4.0 mg/kg), and butorphanol (5.0 mg/kg). Blood samples were collected to measure the concentrations of serum Ca, Pi, parathyroid hormone (PTH), serum creatinine (CRE), and blood urea nitrogen (BUN).

The descending aorta, extending from the thoracic to the abdominal region distal to the branching point of the subclavian artery, was harvested and used for analysis. The aorta was divided into two regions: (1) a segment approximately 5 mm distal to the subclavian artery (defined as the highly calcified region), and (2) the remaining distal portion (defined as the mildly calcified region) (Supplementary Fig. 1).

For each experimental group (*n* = 12), mice were subdivided for histological analyses (*n* = 6) and gene expression analyses (*n* = 6). For histological analyses, the highly calcified region was further subdivided into upper and lower portions for histochemical analysis and transmission electron microscopy–energy-dispersive X-ray spectroscopy (TEM-EDX), respectively. The mildly calcified region was subdivided into three segments for histochemical analysis, TEM, and immunoelectron microscopy. For gene expression analyses, highly calcified regions were used for reverse transcription–quantitative polymerase chain reaction (RT-qPCR), whereas mildly calcified regions were used for both RT-qPCR and RNA sequencing (RNA-seq).

### Tissue preparation

Aortae from each experimental group (*n* = 6 each) were fixed in 4% paraformaldehyde containing 0.1 M cacodylate buffer (pH 7.4) for 18 h at 4 ℃. The aortae were divided as described above. Specimens for histochemical analysis were immersed in 0.067 M cacodylate buffer (pH 7.4), while those for TEM were fixed in 2% paraformaldehyde and 2.5% glutaraldehyde containing the same buffer, and the specimens were incubated for 24 h at 4 ℃. Some of the fixed aortae were decalcified in 5% ethylenediaminetetraacetic acid disodium salt solution, while the others remained undecalcified. For ultrastructural observation, aortae were post-fixed in 1% osmium tetroxide containing 0.1 M cacodylate buffer (pH 7.4). Samples of both decalcified and undecalcified aortae were dehydrated and embedded as follows: in a graded ethanol series and paraffin for histochemical analyses; in a graded acetone series and epoxy resin for ultrastructural observation using TEM (Hitachi 7800; Hitachi Co., Tokyo, Japan); or in a graded N, N-dimethylformamide series and glycidyl methacrylate (GMA) for immunoelectron microscopy.

### Immunohistochemistry

Paraffin-embedded decalcified sections were deparaffinized, rinsed in phosphate-buffered saline (PBS), and treated with 0.3% hydrogen peroxide for 30 min. Nonspecific binding was blocked with 1% bovine serum albumin (BSA; Seologicals Proteins Inc., Kankakee, IL, USA) in PBS. Immunostaining was performed using the primary and secondary antibodies listed in Supplementary Table 1, as previously described^33,34^. Immunoreactivity was visualized using 3,3′-diaminobenzidine tetrahydrochloride (Dojindo Laboratories, Kumamoto, Japan).

For the double detection of α-smooth muscle actin (αSMA) and matrix Gla protein (MGP), dewaxed paraffin sections were blocked with 1% BSA in PBS, incubated with a mouse anti-αSMA antibody, and subsequently with a horseradish peroxidase (HRP)-conjugated secondary antibody. After visualizing the HRP reaction, the immunostained sections were incubated with an anti-MGP antibody, followed by an ALP-conjugated secondary antibody. The ALP reaction was visualized by immersing sections in 30 mL of 0.1 M Tris-HCl buffer (pH 8.5) containing 2.5 mg of naphthol AS-BI phosphate (Sigma-Aldrich Co., LLC., St. Louis, MO) and 18 mg of Fast Blue RR salt (Sigma-Aldrich) at 37 ℃ for 15 min, until immunoreactivity was evident.

For the co-detection of calcified matrix and MGP, paraffin-embedded undecalcified sections were processed for MGP immunohistochemistry as described above. The stained sections were rinsed with PBS and treated with a 5% aqueous silver nitrate solution, as described below. All sections were counterstained with methyl green and examined under a light microscope (Eclipse Ni; Nikon Instruments Inc., Tokyo, Japan).

### Van Gieson staining

To stain elastic fibers, paraffin-embedded undecalcified sections were treated with 37 mL of Weigert’s resorcin-fuchsin solution for 1 h. The staining solution was prepared by mixing 3.4 mL of resorcin–fuchsin stock solution containing 29.6 mg of basic fuchsin (FUJIFILM Wako Pure Chemical Corporation, Osaka, Japan), 74.3 mg of resorcinol (Sigma-Aldrich), and 0.11 mg of iron(III) chloride (FUJIFILM Wako Pure Chemical Corporation) with 33 mL of 70% ethanol and 0.6 mL of hydrochloric acid. Subsequently, the sections were rinsed with distilled water, immersed in iron hematoxylin for 5 min, and stained with van Gieson solution consisting of saturated picric acid solution (Sigma-Aldrich) containing 1% acid fuchsin (FUJIFILM Wako Pure Chemical Corporation) for 10 min.

### Alizarin red staining and von Kossa staining

Aortas were stained with 1% Alizarin Red S solution (pH 4.2) for 10–20 min at room temperature and rinsed thoroughly with water to remove excess dye. Von Kossa staining was performed as previously described^33^ to detect mineralized matrices. Briefly, paraffin-embedded undecalcified sections and undecalcified semithin epoxy resin sections were incubated in 5% aqueous silver nitrate (FUJIFILM Wako Pure Chemical Corporation) under sunlight for 30 min. The sections were then rinsed with distilled water and incubated in 5% sodium thiosulfate solution (FUJIFILM Wako Pure Chemical Corporation) for 5 min to remove unreacted silver and stabilize staining. Sections were counterstained with methyl green for paraffin-embedded sections or toluidine blue for epoxy resin-embedded sections.

### Enzyme histochemistry for tartrate-resistant acid phosphatase

To detect tartrate-resistant acid phosphatase (TRAP) activity, paraffin-embedded decalcified sections were rinsed with PBS and incubated in 30 mL of 0.1 M sodium acetate buffer (pH 5.0) containing 2.5 mg naphthol AS-BI phosphate (Sigma-Aldrich), 18 mg red violet LB (Sigma-Aldrich) salt, and 0.76 g L-(+)-tartaric acid (100 mmol/L) for 15 min at 37 ℃, as previously described^33^. All sections were counterstained with methyl green.

### Quantification of the calcified area in the aorta

Thirty cross-sections were prepared at equal intervals from each aorta sample and stained using von Kossa staining. The total cross-sectional aortic area and von Kossa-positive calcified area in each cross-section were measured, and the ratio of von Kossa-positive calcified area to total aortic area (%) was calculated.

### Immunoelectron microscopy

Undecalcified GMA-embedded thin aorta sections from *kl/kl* mice were blocked with 1% BSA in PBS, incubated with an anti-MGP antibody, and incubated with a 15 nm gold particle-conjugated secondary antibody. Immunolabeled MGP in the *kl/kl* aorta was subsequently examined using TEM.

### TEM-EDX and selected area electron diffraction

TEM-EDX and selected area electron diffraction were performed on aortae from *kl/kl* mice and femora from *WT* mice to investigate the distribution of Ca and phosphorus (P) in calcified areas. Epoxy resin-embedded undecalcified thin sections were prepared without heavy metal staining to avoid diffraction pattern interference. Elemental analysis was conducted using a TEM equipped with an EDX detector (JEM-1400; JEOL Ltd., Tokyo, Japan) operated at an accelerating voltage of 80 kV. EDX spectra were acquired from selected regions of interest in scanning TEM mode. Quantitative elemental compositions were determined using standardless ZAF correction, and elemental maps for Ca and P were generated using EDX software. Selected area electron diffraction patterns were obtained from individual mineral crystallites within the mineralized aorta and bone matrix using selected area apertures.

### Micro-CT analysis

Micro-computed tomography (CT) aorta images were obtained using a micro-CT system (CosmoScan FX, Rigaku Corporation, Tokyo, Japan) at 90 kV and 88 µA, with a 25 mm field of view and 20 μm voxel size. Images were reconstructed using CosmoScan Viewer software (Rigaku Corporation) following the method described by Bouxsein et al.^35^.

### Quantitative RT-qPCR analysis

Aortic tissue samples obtained from the mildly calcified region of *WT*, *WT*-Upa, *kl/kl*, and *kl/kl*-Upa mice, as well as from the highly calcified region of *WT* and *kl/kl* mice (*n* = 6 per group), were homogenized in TRIzol reagent (Life Technologies Co., Carlsbad, CA, USA) for total RNA extraction. The extracted RNA pellets were washed in 75% ethanol, briefly air-dried, and dissolved in DEPC-treated water. First-strand cDNA was synthesized using the SuperScript VILO cDNA Synthesis Kit (Life Technologies Co.). Gene expression levels were measured using a quantitative PCR system (StepOne; Thermo Fisher Scientific Inc., Waltham, MA, USA) and normalized to *Gapdh* expression using the ΔΔCt method. The stability of *Gapdh* expression across experimental groups was evaluated by comparing Ct values. Variability was assessed using standard deviation, and differences among groups were analyzed using one-way analysis of variance (ANOVA) followed by Tukey–Kramer multiple-comparison tests or Welch’s *t*-test. Results of *Gapdh* stability analysis are shown in Supplementary Fig. 2. The examined genes included *Pth1r*,* Slc20a1* (*Pit1*), *Slc20a2* (*Pit2*), *Casr*,* Fgfr1*,* Galnt3*,* Acta2* (*αsma*), *Alpl*,* Phospho1*,* Enpp1*,* Sp7* (*Osterix*), *Runx2*,* Eln* (*Elastin*), *Ctsk* (*Cathepsin k*), *Ctss* (*Cathepsin s*), *Elane* (*Elastase*), *Mmp2*,* Mmp9*,* Spp1* (*osteopontin*), *Mgp*,* Atf4*,* Lrp5*,* Lrp6*,* Wnt1a*,* Wnt3a*,* Bmp2*,* Bmp4*,* Sox9*,* Col2a1*,* Col10a1*,* and Acan*. TaqMan probes (Applied Biosystems, Waltham, MA, USA) used for the analysis are listed in Supplementary Table 2.

### RNA-Seq analysis

Total RNA was extracted from aortic tissues of *WT*, *kl/kl*, and *kl/kl*-Upa mice (*n* = 3 per group) and subjected to RNA-Seq to compare transcriptomes between *WT* and *kl/kl* mice and between *kl/kl* and *kl/kl*-Upa mice. RNA-seq libraries were prepared using the NEBNext Ultra II Directional RNA Library Prep Kit (E7760; New England Biolabs, Tokyo, Japan) and sequenced on a NovaSeq 6000 platform (Illumina, San Diego, CA, USA) to generate 150-bp paired-end reads. Raw reads were trimmed using Trimmomatic (version 0.38) and aligned to the mouse reference genome (GRCm38/mm10) using HISAT2 (version 2.1.0). Read counts were generated using featureCounts (version 1.6.3), and expression levels were normalized as FPKM and TPM values. Differentially expressed genes were identified using DESeq2 (version 1.24.0) with Benjamini–Hochberg correction (|log2FC| > 1, FDR < 0.05). Heatmaps were generated based on normalized expression data.

### Measurement of serum Ca, Pi, PTH, BUN, and CRE

Blood samples were obtained from all mice as previously described^24^. Serum levels of Ca, Pi, PTH, BUN, and CRE were measured by Oriental Yeast Co., Ltd. (Tokyo, Japan) using commercial assay kits according to the manufacturers’ protocols.

### Statistical analysis

Normality and homogeneity of variance were assessed using the Shapiro–Wilk test and Levene’s test, respectively. For pairwise comparisons between two groups, Welch’s *t*-test was used. For comparisons among four groups (*WT*, *kl/kl*, *WT*-Upa, and *kl/kl*-Upa), one-way ANOVA followed by Tukey–Kramer multiple-comparison tests was used when assumptions of normality and homogeneity of variance were met. When these assumptions were not satisfied, non-parametric tests were used. Specifically, the Mann–Whitney *U* test was used for two-group comparisons, and the Kruskal–Wallis test followed by appropriate post hoc comparisons was used for multiple groups. Results are presented as mean ± standard error, and *p* < 0.05 was considered statistically significant.

## Results

### Expression and localization of osteogenic differentiation factors and calcification-related molecules in the aorta of ***kl/kl*** mice

**Fig. 1:**
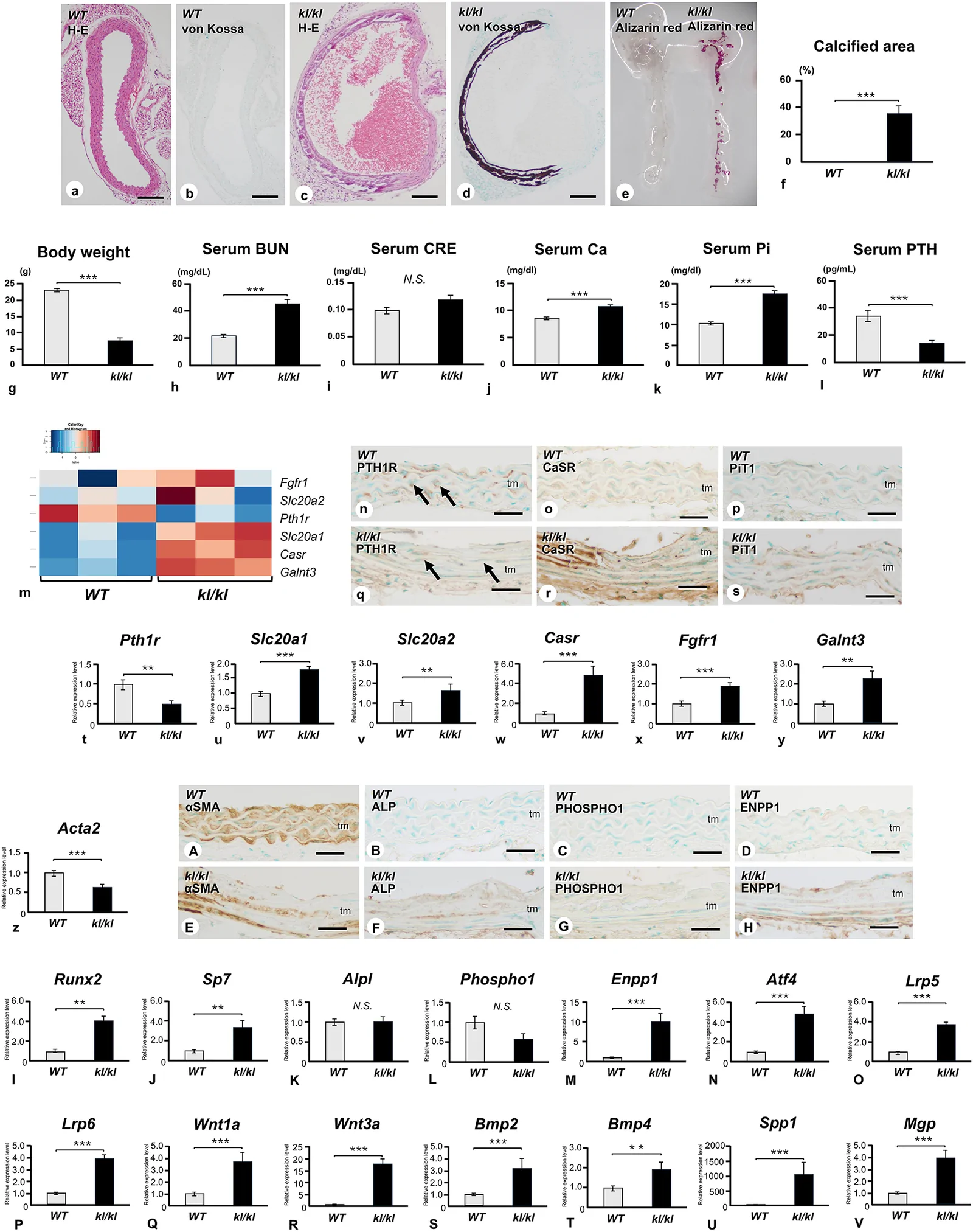
Body weight, serum markers, and distribution of osteogenic markers and calcification-promoting molecules in the mildly calcified region of wild-type (*WT*) and* kl/kl *aorta. **a, c: **Hematoxylin and eosin (H-E) staining images showing aortic cross sections. **b, d: **von Kossa staining images showing aortic cross-sections; black staining indicates mineralized matrices. **e**: Alizarin Red staining images of the aorta; red staining indicates mineralized matrices.** f: **Proportion of calcified area (%) in the aortae of *WT* and *kl/kl* mice. **g: **Body weight (g) of *WT* and *kl/kl* mice. **h–l:** Serum levels of the indicated markers. **m: **Heatmap depicting the expression of calcification-promoting genes in the aortae of *WT *and* kl/kl *mice based on RNA-seq analysis. **n–s:** Immunohistochemistry images showing localization of PTH1R (**n, q**), CaSR (**o, r**), and PiT1 (**p, s**) in aortic tissue. Brown staining indicates positive immunoreactivity in each specimen (“tm” denotes the tunica media). Methyl green was used to counterstain nuclei.** t–y:** Relative gene expression levels of* Pth1r* (**t**), *Slc20a1 *(**u**), *Slc20a2 *(**v**), *Casr *(**w**), *Fgfr1 *(**x**), and *Galnt3 *(**y**), measured by RT-qPCR. **z:** Relative *Acta2* expression, measured by RT-qPCR. **A–H: **Immunohistochemistry images showing αSMA (**A, E**), ALP (**B, F**), PHOSPHO1 (**C, G**), and ENPP1 (**D, H**) localization. **I–V:** Relative gene expression levels of* Runx2 *(**I**)*, Sp7 *(**J**)*, Alpl *(**K**)*, Phospho1 *(**L**)*, Enpp1 *(**M**),* Atf4* (**N**), *Lrp5* (**O**), *Lrp6 *(**P**), *Wnt1a* (**Q**), *Wnt3a* (**R**), *Bmp2 *(**S**), *Bmp4 *(**T**),*Spp1* (**U**), and *Mgp* (**V**) in the aorta of *WT* and *kl/kl* mice, measured by RT-qPCR. All quantitative data are presented as mean ± standard error. Statistical significance was assessed using Welch’s *t-*test; *p*-values < 0.05 were considered significant and are indicated as follows: ***, *p* < 0.001; **,* p* < 0.01; *, *p* < 0.05. Scale bars represent 100 μm (**a–d**) and 30 μm (**n–s, A–H**).

Histological staining revealed extensive calcification throughout the aortic media of *kl/kl* mice **(**Figs. 1a–f**)**. However, calcification was spatially heterogeneous, with regions exhibiting marked Ca/Pi deposition under Alizarin Red staining interspersed with apparently uncalcified areas **(**Fig. 1e**)**. Serum BUN was significantly elevated in *kl/kl* mice **(**Fig. 1h**)**, whereas serum CRE showed no significant difference **(**Fig. 1i**)**. Serum Ca levels were slightly but significantly higher in *kl/kl* mice than in *WT* mice **(**Fig. 1j**)**, and serum Pi levels were approximately 1.8-fold higher in *kl/kl* mice **(**Fig. 1k**)**. In contrast, serum PTH levels were significantly decreased **(**Fig. 1l**)**.

RNA-seq **(**Fig. 1m**)** suggested changes in the expression of several genes, which were subsequently validated by RT-qPCR analyses **(**Fig. 1t–y**)**. Specifically, RT-qPCR demonstrated that expression of the PTH receptor gene *Pth1r* was reduced in the mildly calcified regions of the *kl/kl* aorta **(**Fig. 1t**)**, whereas expression of the phosphate transporter genes *Slc20a1 (Pit1)* and *Slc20a2 (Pit2)*, the *CaSR* gene *Casr*, and the genes *Fgfr1* and *Galnt3* were significantly elevated **(**Fig. 1u–y**)**.　In the aortic media, *kl/kl* mice exhibited reduced *Acta2* (*αsma*) expression and reduced αSMA immunoreactivity compared with *WT* mice **(**Figs. 1z, A, E**)**. In contrast, several osteogenic differentiation–related genes, including *Runx2*, *Sp7* (*Osterix*), *Atf4*,* Lrp5*,* Lrp6*,* Wnt1a*,* Wnt3a*,* Bmp2*,* Bmp4*,* Spp1*,* and Mgp*, were upregulated **(**Figs. 1I, J, N–V**)**. Expression and localization of *Alpl*/ALP and *Phospho1*/PHOSPHO1 showed no significant changes **(**Figs. 1B, C, F, G, K, L**)**. Notably, *Enpp1* expression and ENPP1 immunoreactivity, an enzyme that generates pyrophosphate, were upregulated **(**Figs. 1D, H, M**)**.

### Ultrastructural features of the mildly calcified region in the aorta of ***kl/kl*** mice

**Fig. 2:**
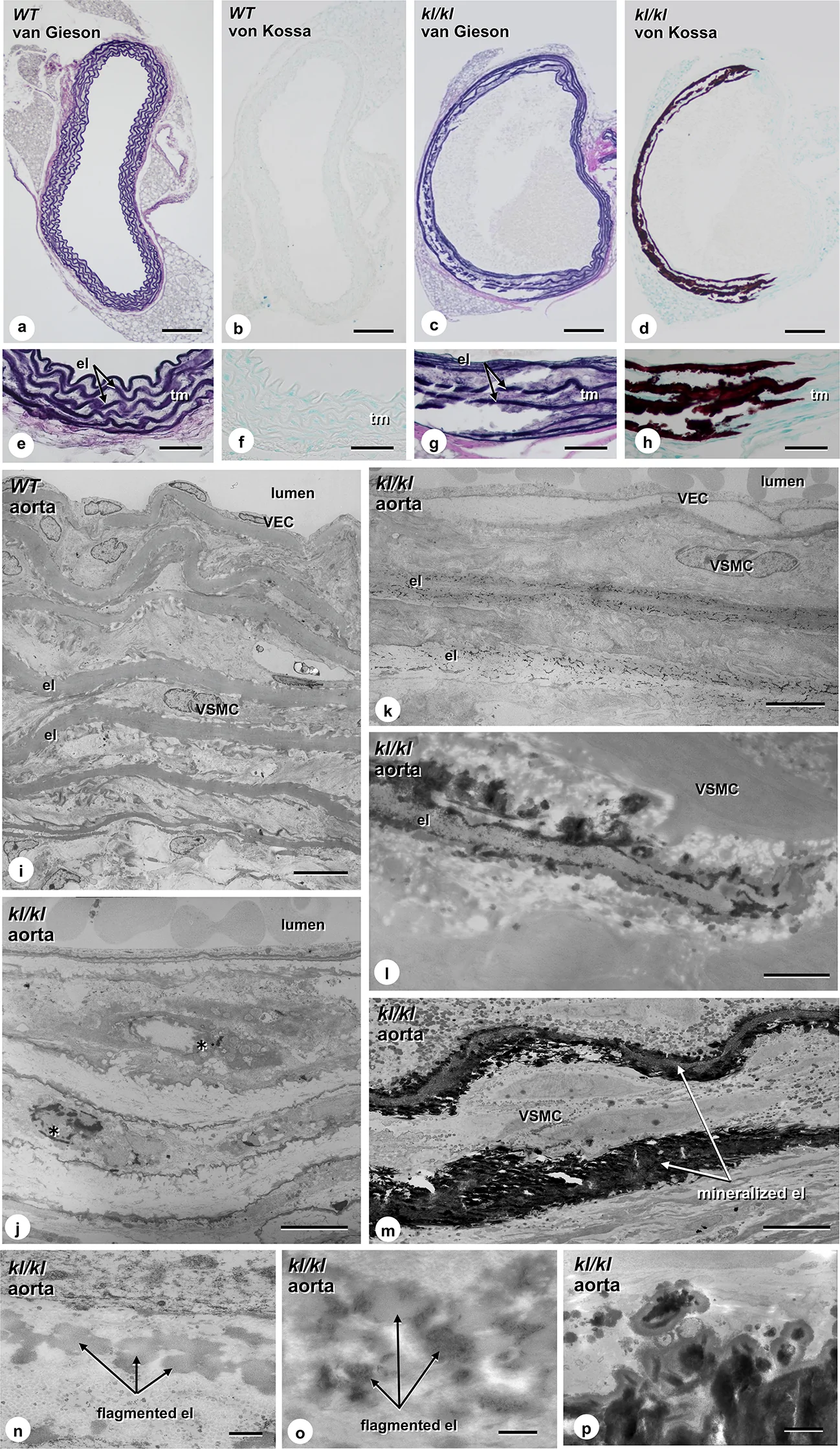
Ultrastructure of the mildly calcified region of the aortae in *WT* and *kl/kl *mice. **a, c, e, g: **van Gieson staining images showing aortic cross-sections from *WT* and* kl/kl* mice. Purple staining represents elastic lamellae. Panels **e **and **g** show higher-magnification images corresponding to panels **a **and **c**, respectively.** b, d, f, h: **von Kossa staining images showing aortic cross-sections. Panels **f **and **h** are high-magnification images corresponding to panels **b **and **d**, respectively. **i: **TEM image of undecalcified aortic tissue from *WT* mice.** j–m:** TEM images of undecalcified aortic tissue from* kl/kl *mice. Asterisks denote dead VSMCs. **n–p:** High magnification TEM images showing fragmented elastic lamellae in *kl/kl *mice. Scale bars: 100 μm (**a–d**), 30 μm (**e–h**), 5 μm (**i–k, m**)**,** 2 μm (**l**), 500 nm (**n**), and 200 nm (**o, p**). Abbreviations: tm, tunica media; el, elastic lamella; VEC, vascular endothelial cell; and VSMC, vascular smooth muscle cell.

In *WT* mice, elastic lamellae in the aortic media exhibited an undulating morphology **(**Figs. 2a, b, e, f**)**. In contrast, elastic lamellae in the mildly calcified regions of the *kl/kl* aorta appeared stretched and straightened, with mineral deposits distributed along their surfaces **(**Figs. 2c, d, g, h**)**. TEM revealed continuous, undulating lamellae in *WT* mice **(**Fig. 2i**)**, whereas numerous minute calcified granules were observed on the surface of elastic lamellae in *kl/kl* mice **(**Fig. 2k**)**. At higher magnification, Ca/Pi deposition was preferentially localized on or adjacent to elastic lamellae rather than in the surrounding interlamellar extracellular matrix containing VSMCs **(**Figs. 2l, m**)**. These findings suggest that elastin-containing lamellae are initial sites of calcification. In addition, VSMCs exhibiting morphological features consistent with apoptotic or degenerative changes were observed in areas enclosed by calcified elastic lamellae **(**Fig. 2j**)**.

To determine whether these calcified structures resemble classical matrix vesicles or calcifying nodules observed in bone, we performed comparative ultrastructural analyses (Supplementary Fig. 3). In *WT* femora, numerous calcifying nodules were observed beneath osteoblasts (Supplementary Fig. 3a). At higher magnification, minute calcified crystals were found within matrix vesicles, and aggregates of needle-like hydroxyapatite crystals were identified (Supplementary Figs. 3b, c). In contrast, the *kl/kl* aortic medial tissue exhibited fragmented elastin-derived structures with irregularly shaped or partially rounded morphology arranged in a series (Fig. 2n). Mineral deposits accumulated within or around these irregular elastin fragments **(**Fig. 2o**)**, and some fragments contained spherical calcified inclusions **(**Fig. 2p**)**. However, most of these mineralized structures lacked a lipid bilayer, a characteristic ultrastructural feature of the plasma membrane observed in authentic matrix vesicles in bone, indicating that they are structurally distinct from classical matrix vesicles.

**Fig. 3:**
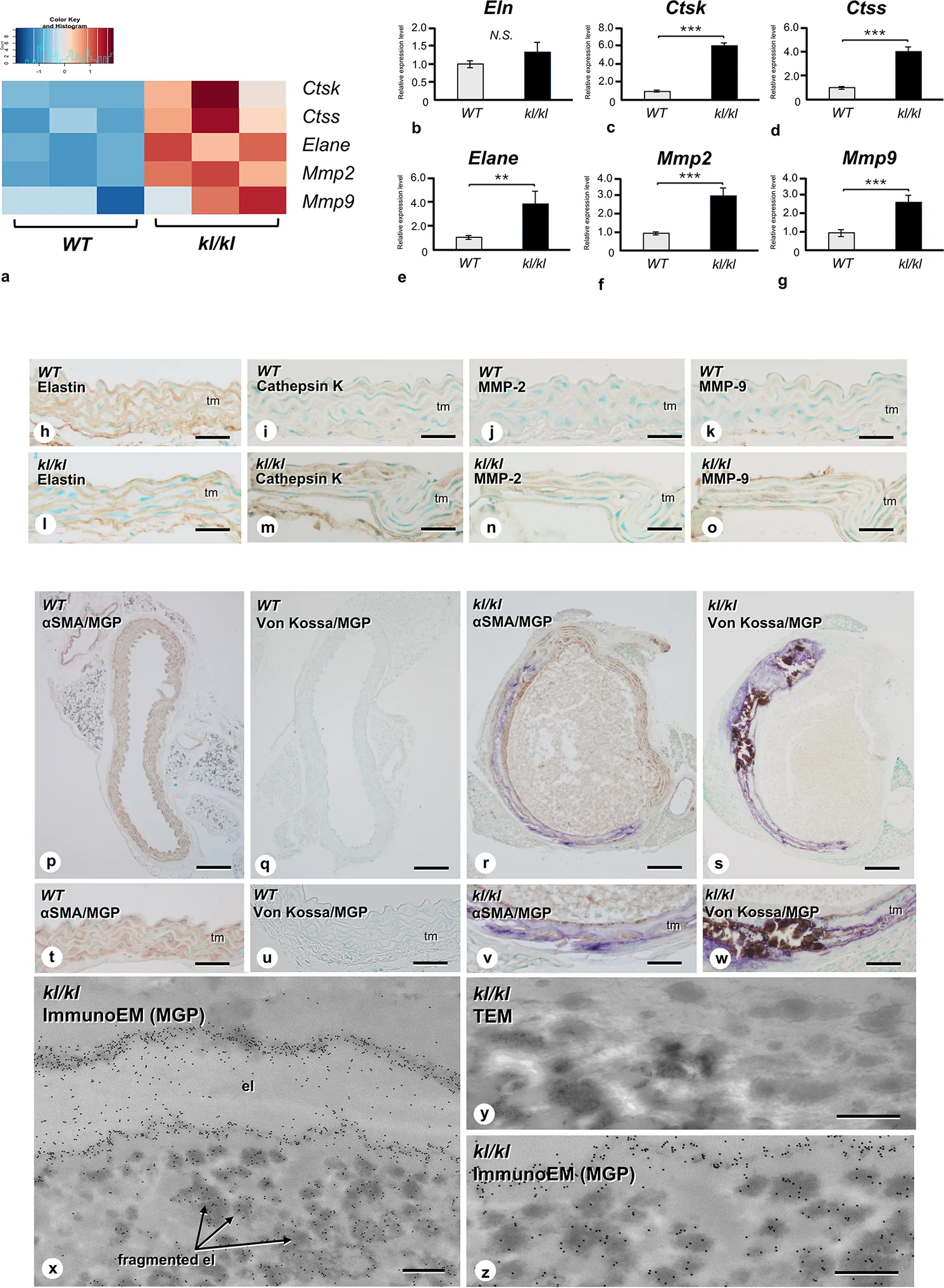
Elastin-degrading enzyme gene expression and localization of elastin-degrading enzymes and matrix Gla protein (MGP) in the mildly calcified region of*** WT ***and* kl/kl* aorta. **a:** Heatmap depicting the expression levels of genes encoding enzymes related to elastin degradation in the aortic tissues, determined using RNA-seq analysis. **b–g: **Relative gene expression levels of *Eln* (**b**),* Ctsk* (**c**), *Ctss *(**d**), *Elane *(**e**), *Mmp2 *(**f**), and *Mmp9* (**g**). **h–o:** Immunohistochemistry images showing elastin (**h, l**), cathepsin K (**i, m**), MMP-2 (**j, n**), and MMP-9 (**k, o**) localization in the aortic tissues. tm, tunica media **p, r, t, v: **Double staining images showing αSMA (brown) and MGP (blue) in aortic cross-sections. Panels **t **and **v** are higher-magnification images corresponding to panels **p **and **r**, respectively. **q, s, u, w:** Double staining images showing von Kossa (black, mineralization) and MGP (blue) in aortic cross-sections. Panels **u **and **w** are higher-magnification images corresponding to panels **q **and **s**, respectively. **x, z:** Immunoelectron microscopy images showing MGP localization in the aortic tunica media from *kl/kl* mice (“el” denotes “elastic lamella”). Dark particles represent gold-labeled MGP. **y:** TEM image of an undecalcified specimen from a region similar to that shown in panel **z**. All quantitative data are presented as mean ± standard error. Statistical significance was assessed using Welch’s *t-*test; *p*-values < 0.05 were considered significant and are indicated as follows: ***, *p* < 0.001, and **,* p*< 0.01. Scale bars: 30 μm (**h–o, t–w**), 100 μm (**p–s**), and 500 nm (**x–z**).

Given the close association between elastin fragmentation and Ca/Pi deposition, we next examined enzymes responsible for elastin degradation. Expression of elastin-degrading proteases was upregulated in *kl/kl* mice, including *Ctsk* and *Ctss*, which encode elastin-degrading cathepsins^18^, as well as *Elane*,* Mmp2*, and *Mmp9*^36^**(**Figs. 3a–g**)**. Immunohistochemistry demonstrated localization of MMP-2, MMP-9, and cathepsin K along stretched elastic lamellae **(**Figs. 3h–o**)**. MGP, a protein that inhibits vascular calcification^37^, was detected in regions with low αSMA immunoreactivity and evident calcification **(**Figs. 3p–w**)**. Immunoelectron microscopy localized MGP to the surfaces of elastic lamellae and adjacent small elastin fragments **(**Figs. 3x–z**)**. These findings suggest that elastin degradation and fragmentation are closely associated with sites of Ca/Pi deposition and the local accumulation of calcification-regulatory proteins.

### Advanced vascular calcification is associated with bone-like tissue formation

**Fig. 4:**
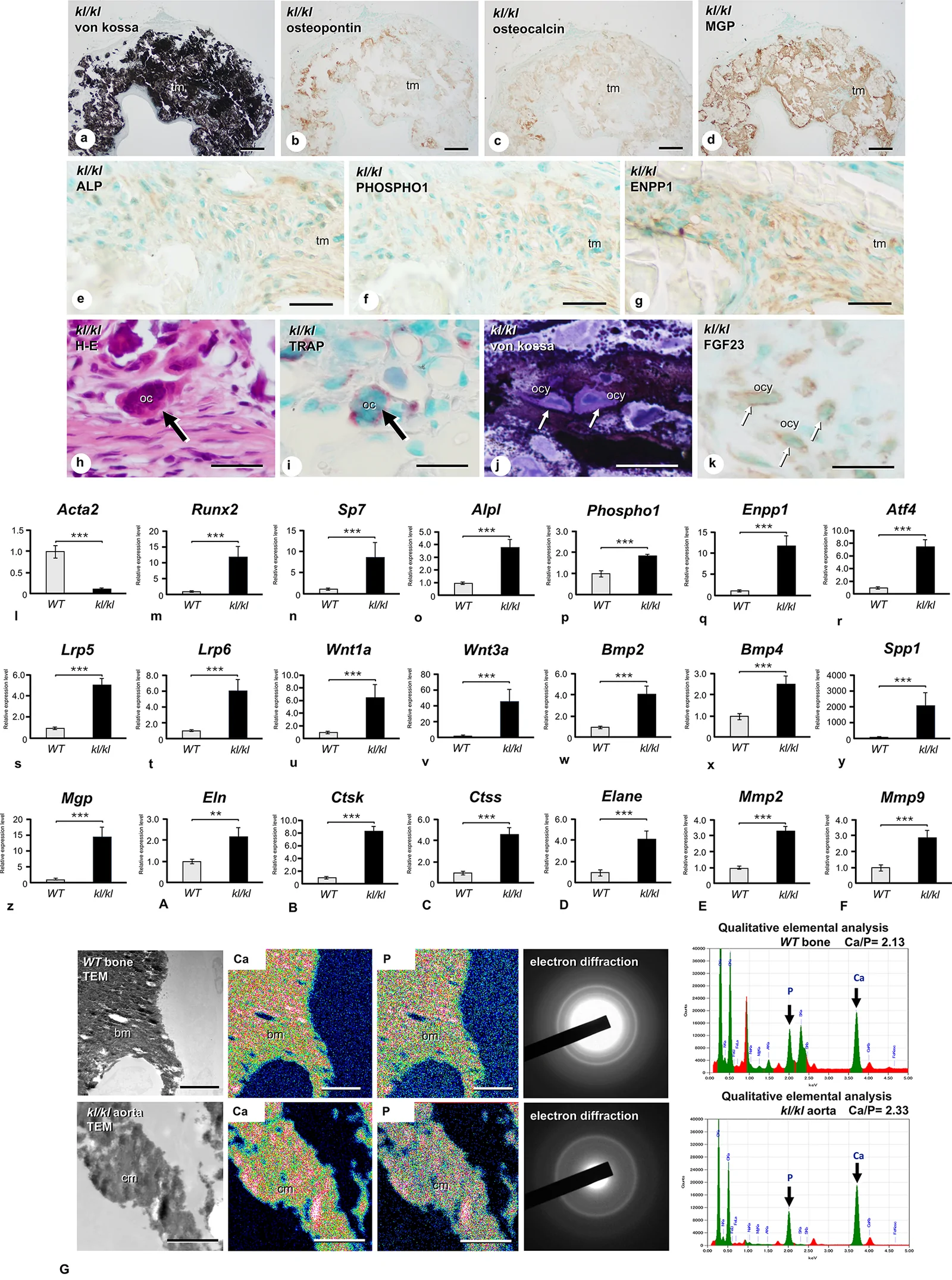
Histopathological features and gene expression changes in the highly calcified aortic region of *kl/kl *mice with advanced vascular ossification. **a–k: **Histopathological images of vascular ossification, including von Kossa staining (**a**; black); immunohistochemistry for osteopontin (**b**), osteocalcin (**c**), MGP (**d**), ALP (**e**), PHOSPHO1 (**f**), and ENPP1 (**g**); H-E staining (**h**), with a black arrow indicating a multinucleated osteoclast-like cell; TRAP staining (**i**; red), with a black arrow indicating a TRAP-positive multinucleated osteoclast-like cell; von Kossa staining of an epoxy resin-embedded semi-thin aorta section (**j**), with white arrows indicating osteocyte-like cells surrounding calcified matrices; and immunohistochemistry for FGF23 (**k**; brown), with white arrows indicating FGF23-positive osteocyte-like cells. **l–F:** Relative gene expression levels of osteogenic differentiation factors and elastin-degrading enzymes in highly calcified regions of *WT *and *kl/kl* mice, including *Acta2 *(**l**), *Runx2*(**m**)*, Sp7 *(**n**)*, Alpl *(**o**)*, Phospho1 *(**p**)*, Enpp1 *(**q**),* Atf4* (**r**), *Lrp5* (**s**), *Lrp6 *(**t**), *Wnt1a* (**u**), *Wnt3a* (**v**), *Bmp2 *(**w**), *Bmp4 *(**x**), *Spp1* (**y**), *Mgp* (**z**), *Eln* (**A**),* Ctsk* (**B**), *Ctss *(**C**), *Elane *(**D**), *Mmp2 *(**E**), and *Mmp9* (**F**).** G:** TEM-EDX and electron diffraction analysis of bone tissue from *WT* mice (upper) and aortic tissue from *kl/kl* mice (lower). All quantitative data are presented as mean ± standard error. Statistical significance was assessed using Welch’s *t-*test; *p* < 0.05 was considered significant (***, *p* < 0.001 and **,* p*< 0.01). Scale bars: 100 μm (**a–d**), 30 μm (**e–g**), 20 μm (**h–i**),10 μm (**j–k**), 5 μm (**x**, upper panels), and 2 μm (**x**, lower panels). Abbreviations: tm, tunica media; oc, osteoclast; ocy, osteocyte; bm, bone matrix; and cm, calcified matrix.

To determine whether advanced calcification involves osteogenic differentiation, we analyzed highly calcified regions of the *kl/kl* aortic media **(**Fig. 4a**)**. The expression of osteoblast differentiation-related genes, including *Runx2*,* Sp7*,* Atf4*, *Lrp5/6*,* Wnt1a/3a*, and *Bmp2/4*, increased significantly, with particularly strong upregulation of *Runx2*,* Sp7*, and *Wnt* ligands. Furthermore, the expression of the calcification-associated enzymes, such as *Alpl* and *Phospho1*, as well as bone matrix–related genes including *Spp1* and *Mgp*, was further elevated compared with mildly calcified regions **(**Figs. 1I–V vs. 4 m–z). Consistent with these findings, ALP-, PHOSPHO1-, and ENPP1-positive osteoblast-like cells were identified, together with FGF23-expressing cells and TRAP-positive cells **(**Figs. 4e–k**)**. The calcified matrix showed immunopositivity for osteopontin, osteocalcin, and MGP **(**Figs. 4b–d**)**. Elemental analysis using EDX revealed Ca/P ratios of 2.13 in *WT* bone and 2.33 in the *kl/kl* aorta **(**Fig. 4G**)**, indicating comparable mineral compositions. Collectively, these observations indicate that advanced medial calcification in *kl/kl* mice exhibits features consistent with vascular ossification, including coordinated osteoblast- and osteoclast-like differentiation and bone-like tissue formation. Notably, elastin-degrading enzymes remained upregulated in these regions **(**Figs. 4B–F**)**, consistent with findings in the mildly calcified regions **(**Figs. 3c–g**)**.

### Upacicalcet suppresses aortic calcification in ***kl/kl*** mice

**Fig. 5:**
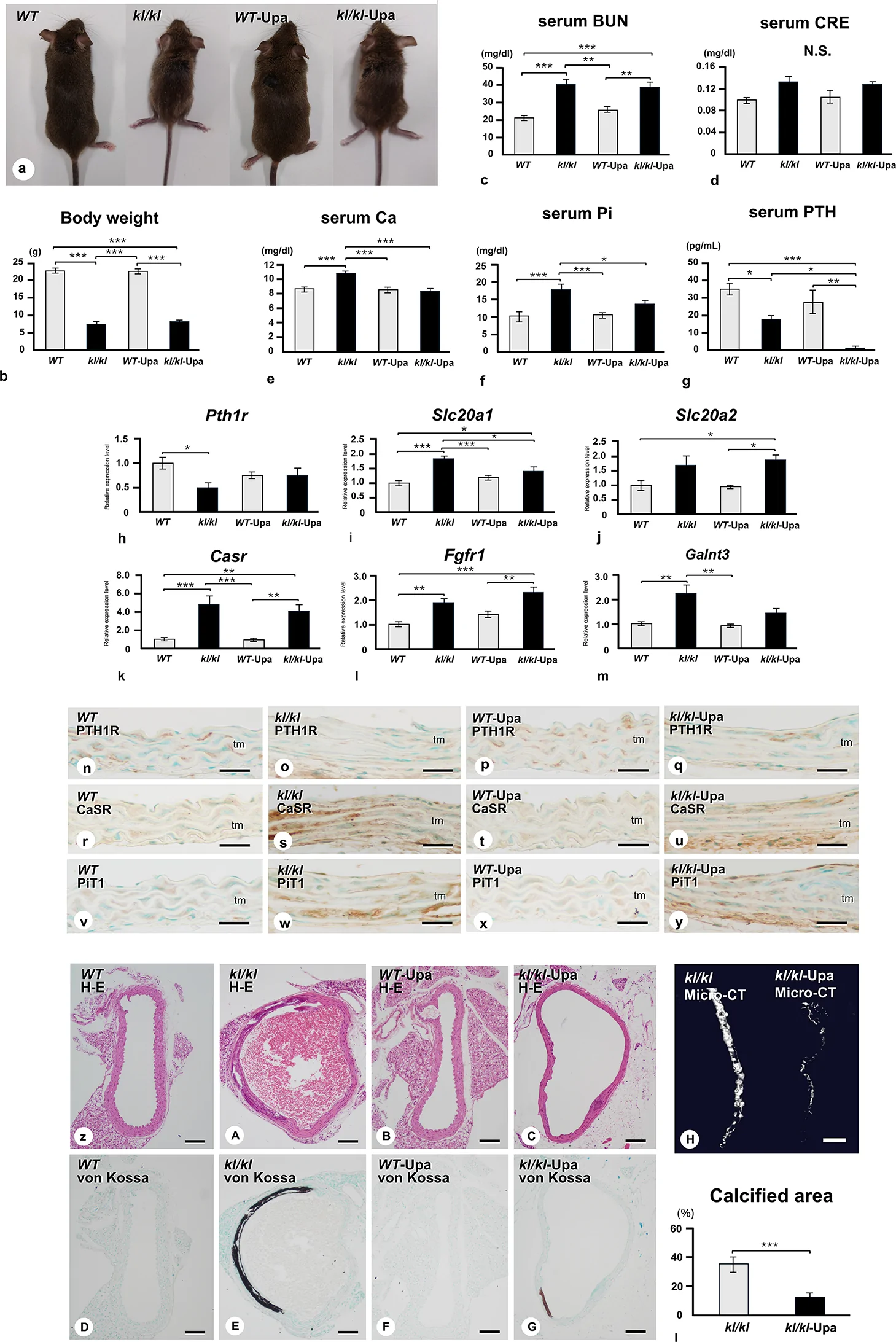
Body weight, serum markers, osteogenic gene expression, and distribution of osteogenic markers and calcification-promoting molecules in the aorta of *WT* and* kl/kl *mice treated with or without upacicalcet (Upa). **a: **Representative whole-body images of *WT*, *kl/kl*,*WT*-Upa, and *kl/kl*-Upa mice.** b:** Body weight (g).** c–g: **Serum levels of indicated markers. **h–m:** Relative gene expression levels of* Pth1r* (**h**), *Slc20a1 *(**i**), *Slc20a2 *(**j**), *Casr *(**k**), *Fgfr1 *(**l**), and* Galnt3 *(**m**).** n–y:** Immunohistochemistry images showing localization of PTH1R (**n–q**), CaSR (**r–u**), and PiT1 (**v–y**) in aortic tunica media (tm). **z–C: **H-E staining images of aortic cross-sections. **D–G: **von Kossa staining images of aortic cross-sections. **H:** Micro-CT images of aortae from *kl/kl* and *kl/kl*-Upa mice. **l: **Proportion of calcified cross-sectional area (%) in the aortic tissues of *kl/kl* and *kl/kl*-Upa mice. All quantitative data are presented as mean ± standard error. Statistical significance was assessed using one-way ANOVA followed by Tukey–Kramer multiple-comparison tests (**b–m**) or Welch’s *t-*test (**l**); *p* < 0.05 was considered significant (***, *p* < 0.001; **,* p* < 0.01; and *, *p* < 0.05). Scale bars: 30 μm (**n–y**), 100 μm (**z–G**), and 2 μm (**H**).

Given prior reports demonstrating anti-calcific effects of calcimimetics^31,38^, we examined the effects of upacicalcet in *kl/kl* mice **(**Fig. 5a**)**. Body weight did not differ between *kl/kl*-Upa and untreated *kl/kl* mice **(**Fig. 5b**)**. Serum CRE levels exhibited no significant differences across all groups **(**Fig. 5d**)**, whereas BUN levels were elevated in both *kl/kl* groups compared with *WT* mice **(**Fig. 5c**)**. *Kl/kl*-Upa mice showed significantly lower serum Ca and PTH levels than *kl/kl* mice (Ca: 8.62 ± 0.29 vs. 10.87 ± 0.29 mg/dL; *p* < 0.001; PTH: 1.00 ± 1.00 vs. 17.53 ± 2.04 pg/mL; *p* < 0.05) **(**Figs. 5e, g**)**. Serum Pi concentrations were also significantly lower in *kl/kl*-Upa mice than in *kl/kl* mice (13.82 ± 0.98 vs. 17.97 ± 1.46 mg/dL; *p* < 0.05) **(**Fig. 5f**)**. No significant differences were observed between *kl/kl*-Upa and *kl/kl* mice in *Pth1r*, *Slc20a2*,* Casr*, *Fgfr1*, or *Galnt3*. However, *Slc20a1* expression was slightly but significantly reduced in *kl/kl*-Upa mice **(**Figs. 5h–y**)**. Morphometric analysis demonstrated a significant reduction in calcified area in *kl/kl*-Upa mice (13.60 ± 2.33%) compared with untreated *kl/kl* mice (35.70 ± 5.06%; *p* < 0.001) **(**Figs. 5z–I**)**, indicating substantial attenuation of vascular calcification.

### Upacicalcet ameliorates elastin abnormalities in the aorta of ***kl/kl*** mice

**Fig. 6:**
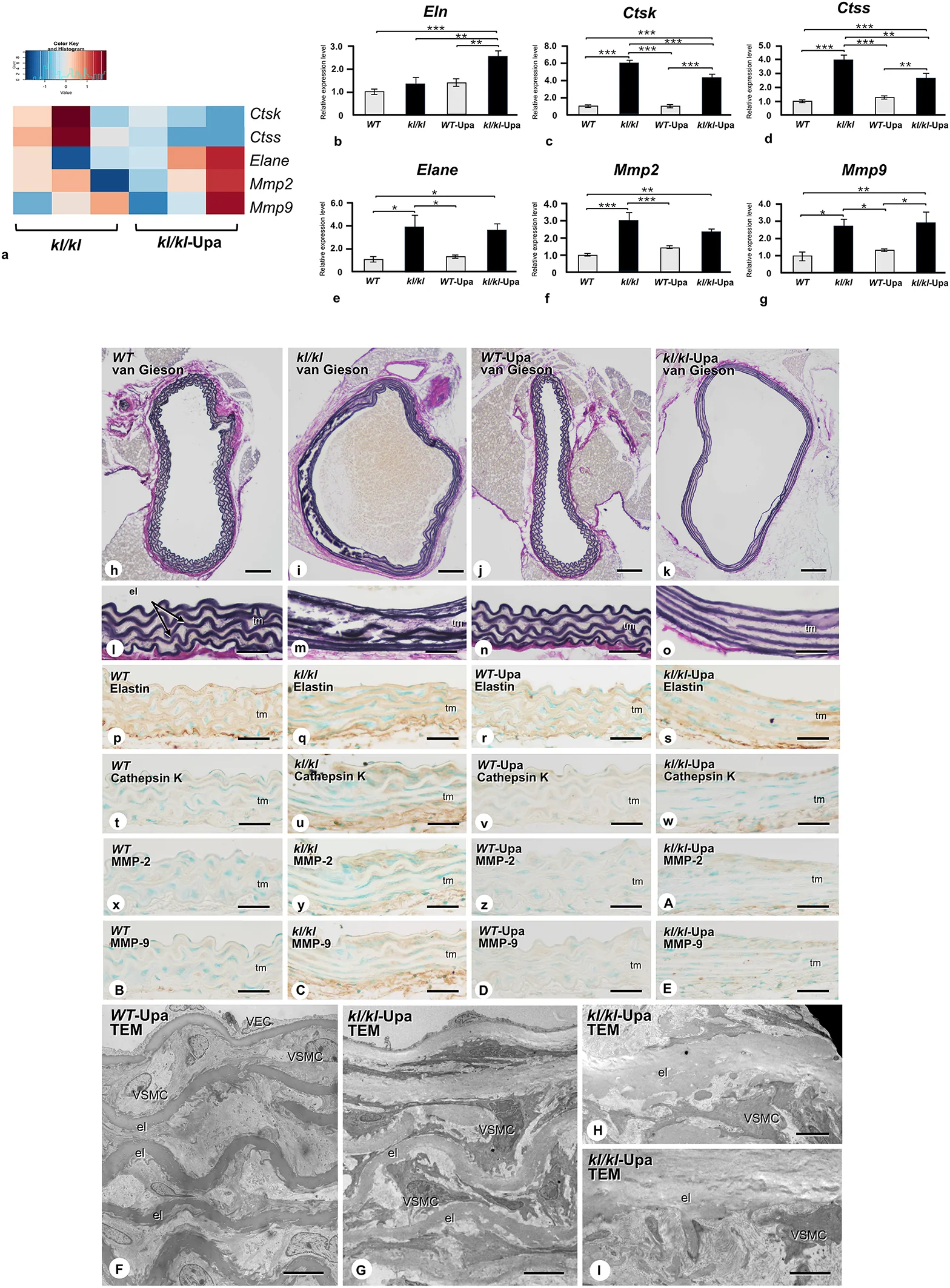
Expression and localization of elastin-degrading enzymes and ultrastructural features of elastic lamellae in the aorta of *WT* and* kl/kl *mice treated with or without Upa. **a:**Heatmap depicting the gene expression levels for enzymes related to elastin degradation in the aortic tissue of *kl/kl* and* kl/kl*-Upa mice, based on RNA-seq. **b–g: **Relative gene expression levels of *Eln* (**b**),* Ctsk* (**c**), *Ctss *(**d**), *Elane *(**e**), *Mmp2 *(**f**), and *Mmp9* (**g**) in aortic tissues. **h–o: **van Gieson staining images of aortic cross-sections. Panels **l–o** show higher-magnification images corresponding to panels. **h–k**, respectively. Purple staining indicates elastic lamellae. **p–E:** Immunohistochemistry images showing localization of elastin (**p–s**), cathepsin K (**t–w**), MMP-2 (**x–A**), and MMP-9 (**B–E**) in aortic tissues. **F–I: **TEM images of undecalcified aortic tissue from *WT*-Upa (**F**) and* kl/kl*-Upa (**G–I**) mice. Panels **H** and **I **are high-magnification TEM images of aortic tissue from *kl/kl*-Upa mice. All quantitative data are presented as mean ± standard error. Statistical significance was assessed using one-way ANOVA followed by Tukey–Kramer multiple-comparison tests; *p* < 0.05 was considered significant (***, *p* < 0.001; **,* p* < 0.01; and *, *p* < 0.05). Scale bars: 100 μm (**h–k**), 30 μm (**l–E**), 5 μm (**F, G**), and 2 μm (**H, I**). tm, tunica media; el, elastic lamella; VEC, vascular endothelial cell; and VSMC, vascular smooth muscle cell.

Given the preferential localization of calcification along elastic lamellae, we analyzed the aortic lamellae in *kl/kl*-Upa mice. *Kl/kl* mice exhibited markedly stretched elastic lamellae with a disorganized and irregular arrangement **(**Figs. 6i, m**)**, whereas in upacicalcet-treated *kl/kl* mice, the elastic lamellae remained stretched but were arranged in a more parallel and orderly manner **(**Figs. 6k, o**)**. RT-qPCR analysis demonstrated increased *Eln* expression and reduced expression of the elastin-degrading enzymes *Ctsk* and *Ctss* in *kl/kl*-Upa mice compared with untreated *kl/kl* mice **(**Figs. 6b–d**)**. *Mmp2* expression showed a decreasing trend, although the difference was not statistically significant **(**Fig. 6f**)**, while *Elane* and *Mmp9* expression were unchanged **(**Figs. 6e, g**)**.

**Fig. 7:**
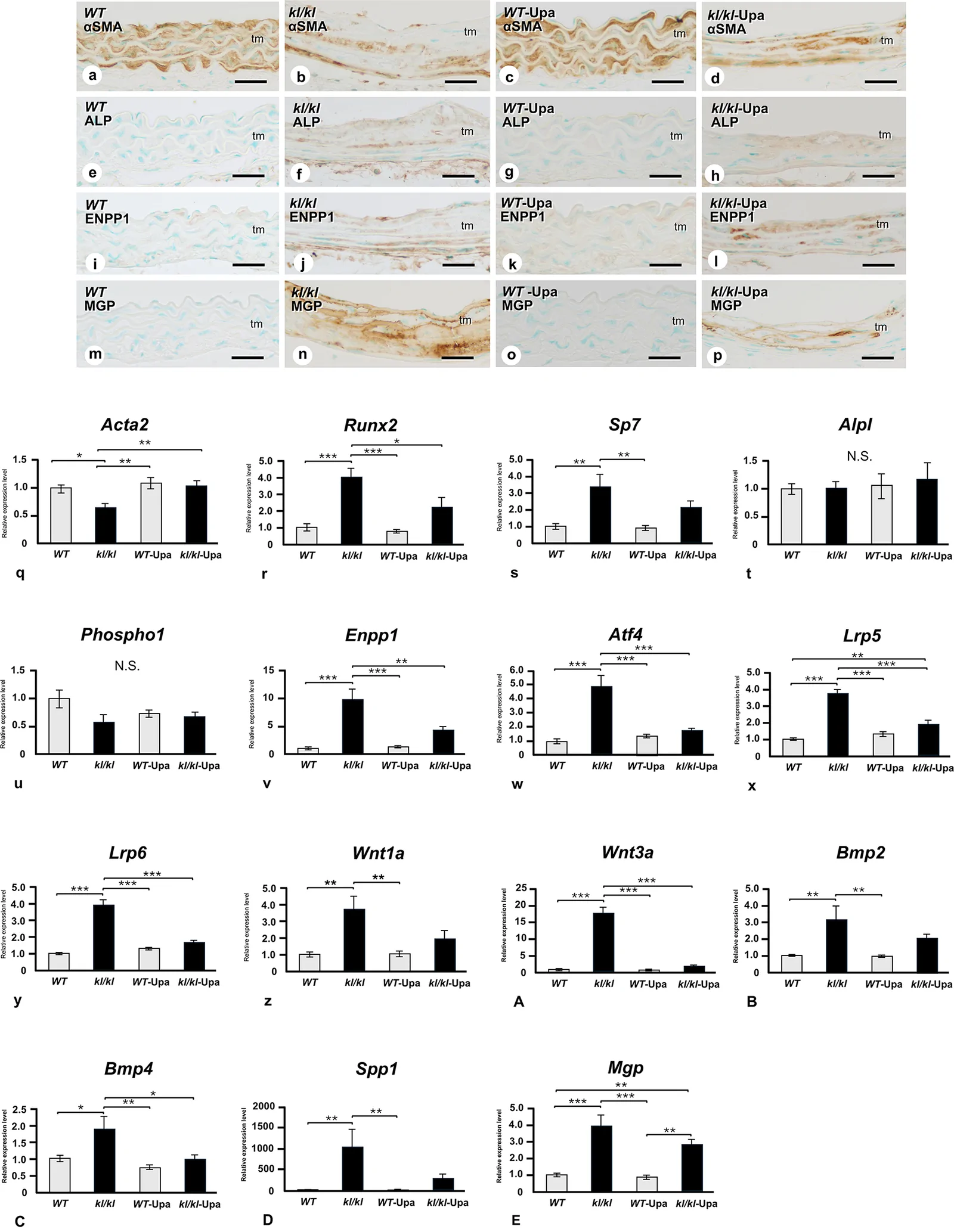
Osteogenic gene expression and protein localization in the aortic tunica media of *WT* and* kl/kl *mice treated with or without Upa. **a–p: **Immunohistochemistry images showing αSMA (**a–d**), ALP (**e–h**), ENPP1 (**i–l**), and MGP (**m–p**) localization (“tm” denotes “tunica media”). **q–E:** Relative gene expression levels of* Acta2 *(**q**), *Runx2 *(**r**)*, Sp7 *(**s**)*, Alpl *(**t**)*, Phospho1 *(**u**)*, Enpp1 *(**v**),* Atf4* (**w**), *Lrp5* (**x**), *Lrp6 *(**y**), *Wnt1a* (**z**), *Wnt3a* (**A**), *Bmp2 *(**B**), *Bmp4 *(**C**), *Spp1* (**D**), and *Mgp* (**E**), measured by RT-qPCR. All quantitative data are presented as mean ± standard error. Statistical significance was assessed using one-way ANOVA followed by Tukey–Kramer multiple-comparison tests; *p* < 0.05 was considered significant (***, *p* < 0.001; **,* p* < 0.01; and *, *p* < 0.05). Scale bars: 30 μm (**a–p**).

*Kl/kl*-Upa mice displayed significantly increased *Acta2* expression **(**Figs. 7a–d, q**)** and significantly reduced *Runx2*,* Enpp1*,* Atf4*,* Lrp5/6*,* Wnt3a*, and *Bmp4* expression **(**Figs. 7r, v, w, x, y, A, C**)**, whereas expression of *Sp7*,* Wnt1a*,* Bmp2*,* Spp1*, and *Mgp* remained unchanged **(**Figs. 7s, z, B, D, E**)**. No significant differences in *Alpl* or *Phospho1* expression were observed among the experimental groups **(**Figs. 7e–h, t, u**)**. Ultrastructural examination of the *kl/kl*-Upa aorta revealed continuous bands of elastic lamellae similar to those observed in *WT*-Upa mice **(**Figs. 6F, G**)**. Although fragmented elastin aggregates were still present, most lacked associated Ca/Pi deposition **(**Figs. 6H, I**)**.

## Discussion

In this study, we analyzed the ultrastructure of the aortic media during the early stages of calcification in *kl/kl* mice and examined the effects of the calcimimetic upacicalcet. Our findings demonstrate that medial vascular calcification is characterized by degeneration and fragmentation of elastin at the surfaces of elastic lamellae, followed by Ca and Pi deposition within these fragmented elastin aggregates. In parallel, osteoblast-like cell differentiation within the tunica media appeared to occur concomitantly with elastin-associated calcification, suggesting that both physicochemical and biological mechanisms contribute to the progression of vascular calcification. Thus, vascular calcification in *kl/kl* mice likely reflects a complex pathological process involving multiple coexisting mechanisms, including elastin-associated mineral nucleation and osteogenic differentiation. Furthermore, upacicalcet not only reduced serum Ca and PTH levels but also significantly decreased serum Pi concentrations in CKD-MBD model *kl/kl* mice. These changes were accompanied by reduced expression of elastin-degrading enzymes, including cathepsins K and S, as well as reduced expression of osteogenic markers such as *Runx2*, *Lrp5*, *Wnt3a*, and *Bmp4*. Consequently, fragmentation of elastic lamellae and subsequent Ca/Pi deposition within elastin aggregates were significantly inhibited. These findings suggest that upacicalcet suppresses vascular calcification, at least in part, by preserving the structural integrity of medial elastic lamellae.

In the aorta of *kl/kl* mice, Ca/Pi deposition was first observed on the surfaces of elastic lamellae, with calcium phosphate crystals accumulating within and around fragmented elastin masses. These ultrastructural features were distinct from osteoblast-derived matrix vesicles or calcifying nodules, as the structures lacked the lipid bilayer membrane typical of matrix vesicles. Although the expression of enzymes essential for matrix vesicle-mediated calcification, such as ALP and PHOSPHO1, was not upregulated in mildly calcified regions of the *kl/kl* aorta, the expression of osteogenic differentiation factors, including *Runx2*, *Sp7*, *Lrp5/6*, *Wnt1a/3a*, and *Bmp2/4*, was elevated. These findings suggest that matrix vesicle-mediated calcification is not the dominant mechanism at early stages and that osteoblastic differentiation progresses in parallel with elastin-associated calcification through trans-differentiation of VSMCs or recruitment of mesenchymal progenitors. In highly calcified regions, elastin-degrading enzymes remained elevated, and bone-like tissue containing cells expressing markers of osteoblast, osteocyte, and osteoclast lineages was observed (Fig. 4). These observations support the notion that vascular calcification may progress toward bone-like tissue formation within the tunica media consistent with previous reports of osteogenic differentiation in vascular tissues^4–6,39^.

In addition, vascular calcification has been reported to involve not only osteoblastic differentiation but also chondrogenic differentiation^40,41^. In this study, the expression levels of several chondrogenic markers, including *Sox9*,* Col2a1*,* Col10a1*, and *Acan*, were significantly increased in the aorta of *kl/kl* mice (Supplementary Fig. 4), suggesting partial activation of chondrogenic differentiation. However, the absence of morphologically identifiable cartilage suggests that this is an incomplete or transitional differentiation state. Therefore, it is likely that osteogenic signaling predominates over chondrogenic pathways in this model. Further detailed investigations are required to clarify these mechanisms.

A key finding of this study is the close association between elastin fragmentation and localized calcification. Elastin fragmentation enhances Ca binding, leading to localized Ca/Pi supersaturation, which in turn promotes hydroxyapatite nucleation^18,42,43^. Consistent with these findings, *kl/kl* mice exhibited increased expression of cathepsins K and S and MMP-2 and -9, concurrent with elastic lamella fragmentation and progressive mineral accumulation. These findings support the idea that elastolytic enzymes are key drivers of early vascular calcification. Moreover, Song and Cerruti^20^ reported that Ca chelation by carboxyl groups derived from degraded elastin and smaller elastin fragment sizes promote Ca/Pi crystal nucleation. The presence of Ca/Pi deposition within fragmented elastin aggregates observed in our study is consistent with these findings. Additionally, the upregulation of cathepsins and MMPs under hypercalcemic and hyperphosphatemic conditions may create a positive feedback loop that accelerates elastin degradation and calcification^44^.

The anti-calcific effects of calcimimetics in CKD models with hyperparathyroidism have been reported^29–31^. The present study provides new insights into the mechanisms underlying the inhibitory effects of upacicalcet on vascular calcification in *kl/kl* mice, a CKD model characterized by low PTH levels. Regarding renal function parameters, serum BUN was significantly elevated in *kl/kl* mice (Fig. 1h), whereas serum CRE exhibited no significant difference (Fig. 1i). The association between reduced *klotho* expression and impaired renal function has been reported in several studies^22,45^; however, in this study, serum BUN and CRE levels showed discordant patterns in *kl/kl* mice. Given that serum CRE levels are proportional to muscle mass and that *kl/kl* mice had lower body weight than *WT* mice (Fig. 1g), serum CRE may not accurately reflect the degree of renal dysfunction in *kl/kl* mice.

In CKD-associated vascular calcification, serum Ca × Pi concentration is considered an important determinant of Ca/Pi crystal formation^46,47^. Under hypercalcemia or hyperphosphatemia conditions, Ca/Pi crystals may form readily, with elastin serving as a nucleation scaffold, as previously suggested^19,48^. Systemically, upacicalcet decreased serum Ca and Pi levels in *kl/kl* mice, thereby reducing Ca/Pi accumulation and potentially attenuating passive Ca/Pi deposition, particularly on degenerated elastic lamellae. However, these effects cannot be fully explained by systemic mineral changes alone. Upacicalcet may also exert local effects by directly modulating VSMCs to suppress elastin-degrading enzymes and osteogenic markers, potentially through CaSR-mediated pathways. Consistent with this possibility, Hénaut et al.^49^ reported that calcimimetics increase CaSR expression and suppress Ca²⁺-induced calcification in VSMCs, while Engeßer et al.^50^ documented ERK1/2-mediated anti-apoptotic signaling following CaSR activation. Although upacicalcet did not increase CaSR expression in our study, its CaSR-dependent effects cannot be excluded. Taken together, these findings support a dual mechanism involving systemic regulation of mineral metabolism and local modulation of vascular cell responses. This framework may help explain the anti-calcific effects of upacicalcet even under conditions of low PTH levels and reduced bone turnover.

Previous studies in *kl/kl* mice have demonstrated that agents such as the selective mineralocorticoid receptor antagonist eplerenone, magnesium (Mg), and ammonium nitrate suppress vascular calcification without major changes in systemic mineral parameters^28,51,52^. In eplerenone-treated *kl/kl* mice, vascular calcification was suppressed despite no changes in serum 1,25(OH)₂D₃, FGF23, Ca, or Pi levels; notably, aortic expression of PiT1, TNFα, Runx2, Osterix, and ALP was reduced^28^. Similarly, Mg supplementation in *kl/kl* mice suppressed vascular calcification even though serum PTH, 1,25(OH)₂D₃, and Ca levels did not decrease, while serum Pi and FGF23 levels increased. Mg supplementation also reduced the expression of Runx2 and MGP in the aorta, along with genes associated with inflammation and matrix degradation^52^. Ammonium nitrate has likewise been reported to suppress vascular calcification without significantly altering serum levels of active 1,25(OH)₂D₃, Ca, or Pi. Instead, it converts respiratory acidosis to metabolic acidosis and reduces blood pCO₂ and HCO₃⁻ levels^51^. Collectively, these agents suppress vascular calcification without major changes in systemic mineral metabolism, indicating that mechanisms beyond serum Ca and Pi are involved and warrant further investigation of upacicalcet.

This study has certain limitations. First, the relative contributions of elastin-associated mineral nucleation, osteogenic differentiation, and systemic mineral metabolism to vascular calcification remain unclear, and key regulators such as FGF23 and 1,25(OH)₂D₃ were not evaluated and require further investigation. Second, *kl/kl* mice are a severe model of mineral metabolism disorder, and the applicability of these findings to human CKD-associated vascular calcification remains uncertain. Third, although ultrastructural analyses support elastin-mediated mineral nucleation, a causal relationship between elastin degradation and calcification has not been established. Further studies, including inhibition of elastolytic enzymes and lineage tracing approaches, are needed to clarify these mechanisms.

## Conclusion

Our findings demonstrate that aortic calcification in *kl/kl* mice is a multifactorial process involving passive Ca/Pi deposition on degenerated elastic lamellae and biologically regulated calcification associated with osteoblast-like differentiation and vascular ossification. Elastin fragmentation appears to provide a structural scaffold for initial Ca/Pi deposition, while osteogenic signaling drives progression toward bone-like tissue formation. Upacicalcet significantly attenuated vascular calcification, accompanied by reduced elastin degradation and suppression of osteogenic gene expression, likely mediated through systemic modulation of mineral metabolism and local regulation of vascular cell responses. Despite low PTH levels, *kl/kl* mice developed hypercalcemia- and hyperphosphatemia-associated medial calcification resembling human CKD-MBD, supporting their value as an* in vivo *model. Collectively, these findings highlight the importance of preserving elastin integrity and limiting osteogenic reprogramming and suggest that CaSR agonists such as upacicalcet exert anti-calcific effects through structural and cellular mechanisms, providing a framework for therapeutic development.

## Supplementary Information

Below is the link to the electronic supplementary material.

Supplementary Material 1

## Data Availability

The datasets generated and/or analysed during the current study are available in the Gene Expression Omnibus (GEO) repository, GSE315133.
