# Supplementary material for: Elastin calcification during the initial stage of vascular calcification in kl/kl mice is suppressed by the calcimimetic upacicalcet
Source: Sci Rep. 2026 Jun 18;16:27984. doi: 10.1038/s41598-026-56029-z (PMC13550462; doi:10.1038/s41598-026-56029-z)
Supplement: Supplementary file 1 [file 41598_2026_56029_MOESM1_ESM.pdf]

Region of Interest (ROI)

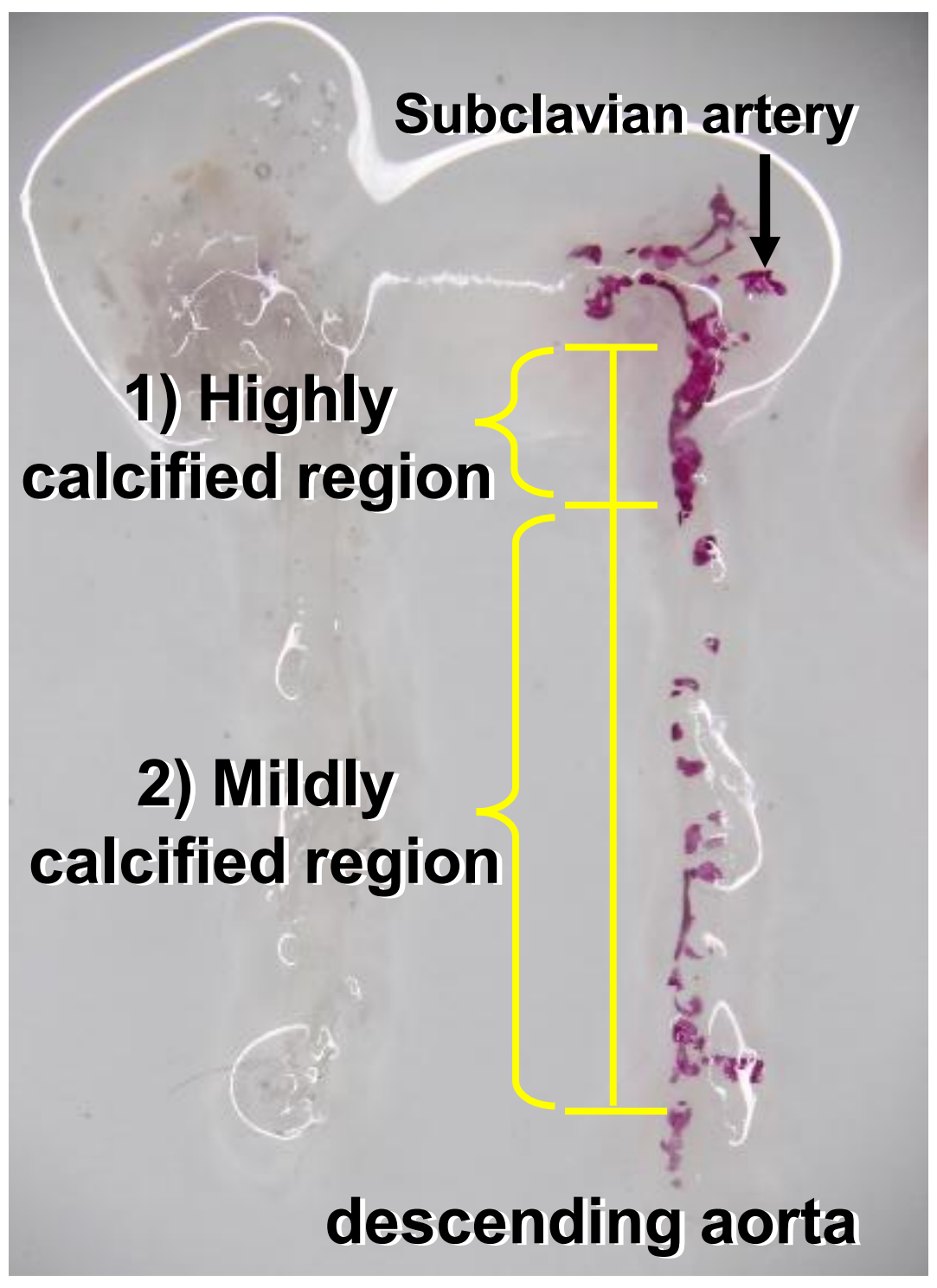

***WT*, *kl/kl*, *WT*-Upa, *kl/kl*-Upa groups**
N = 12 per group

Histological analysis
N = 6 per group

Analytical methods
1) Highly calcified region
Histochemical analysis
TEM–EDX

2) Mildly calcified region:
Histochemical analysis
TEM
Immunoelectron microscopy

Gene expression analysis
N = 6 per group

Analytical methods
1) Highly calcified region:
RT-qPCR analysis

2) Mildly calcified region:
RT-qPCR analysis
RNA-seq analysis

Supplementary Figure 1

primary antibody	cat. No.	company	dilution
Rabbit antibody against PTH/PTHrP receptor	G220	Assay Biotechnology Company, Inc., Sunnyvale, CA	1: 50
Rabbit polyclonal anti-CaSR	PB9924	Boster Biological Technology Co., Ltd., Pleasanton, CA	1: 100
Rabbit polyclonal anti-SLC20A1(Pit1)	GTX105062	GeneTex., Inc., Irvine, CA	1: 100
Mouse antibody against αSMA	MAB1420	R&D systems, Inc., Minneapolis, MN	1: 400
Rabbit polyclonal antisera against TNALPase		Provided by Dr. Oda [52]	1: 300
Rabbit polyclonal anti-PHOSPHO1	Q8TCT1	Cusabio Technology Llc., Houston, TX	1: 100
Goat anti-ENPP1	EB06948	Everest Biotech Ltd., Oxfordshire, UK	1: 200
Rabbit monoclonal anti-Elastin antibody	ab307150	Abcam plc., Cambridge, UK	1: 400
Mouse anti-human cathepsin K	F-95	Kyowa Pharma Chemical Co., Ltd., Takaoka, Japan	1: 100
Rabbit monoclonal anti-hMMP2	F-68	Kyowa Pharma Chemical Co., Ltd., Takaoka, Japan	1: 50
Goat polyclonal anti-mouse MMP9	AF909	R&D Systems, Inc., Minneapolis, MN	1: 100
Rabbit polyclonal anti-Matrix Gla Protein	KR083	Trans Genic Inc., Kobe, Japan	1: 50
Rabbit anti-osteopontin	LB-4225	Cosmo Bio Co., Ltd., Tokyo, Japan	1: 1000
Goat polyclonal anti-osteocalcin	BT-413	Biomedical Technologies Inc., Stoughton, MA	1: 100
Rat monoclonal anti-FGF23	MAB26291	R&D systems, Inc., Minneapolis, MN	1: 100

secondary antibody	cat. No.	company	dilution
HRP-conjugated anti-rabbit IgG	P0399	DakoCytomation, Glostrup, Denmark	1: 100
HRP-conjugated anti-rat IgG	A18921	Zymed Laboratories Inc., South San Francisco, CA	1: 100
HRP-conjugated anti-mouse IgG	61-6520	Chemicon International Inc., Temecula, CA	1: 100
HRP-conjugated anti-goat IgG	A201PS	American Qualex Scientific Products, Inc., San Clemente, CA	1: 100
ALP-conjugated goat Anti-Rabbit IgG	111-055-003	Jackson ImmunoResearch Laboratories, Inc., West Grove, PA	1: 100
EM Goat anti-Rabbit IgG: 20nm Gold	EMGAR20	BBI Solutions., Cardiff, UK	1: 100

Supplementary Table 1

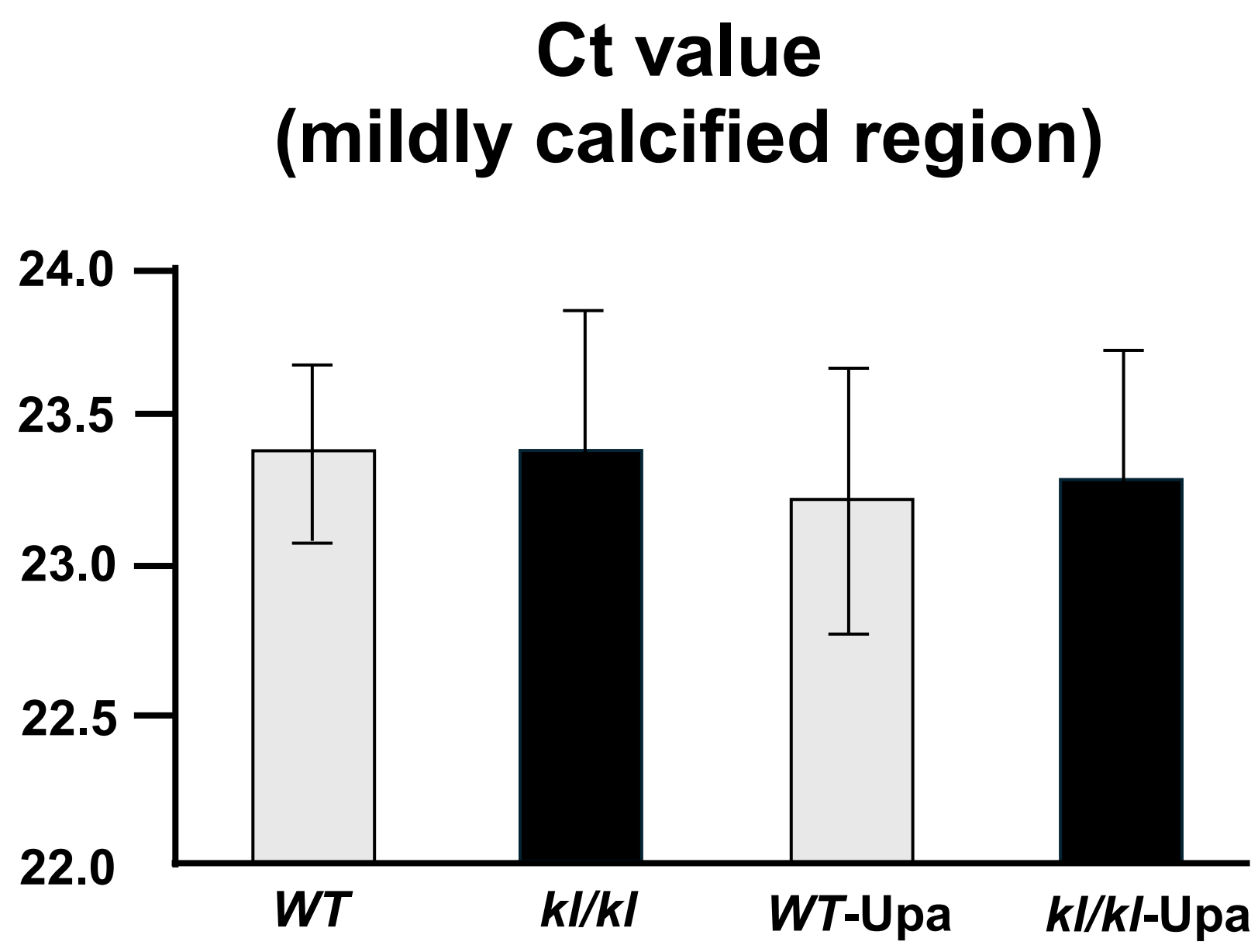

	Ct value	CV (%)
WT	23.38 ± 0.30	1.27
kl/kl	23.40 ± 0.46	1.98
WT-Upa	23.22 ± 0.45	1.93
kl/kl-Upa	23.29 ± 0.44	1.90

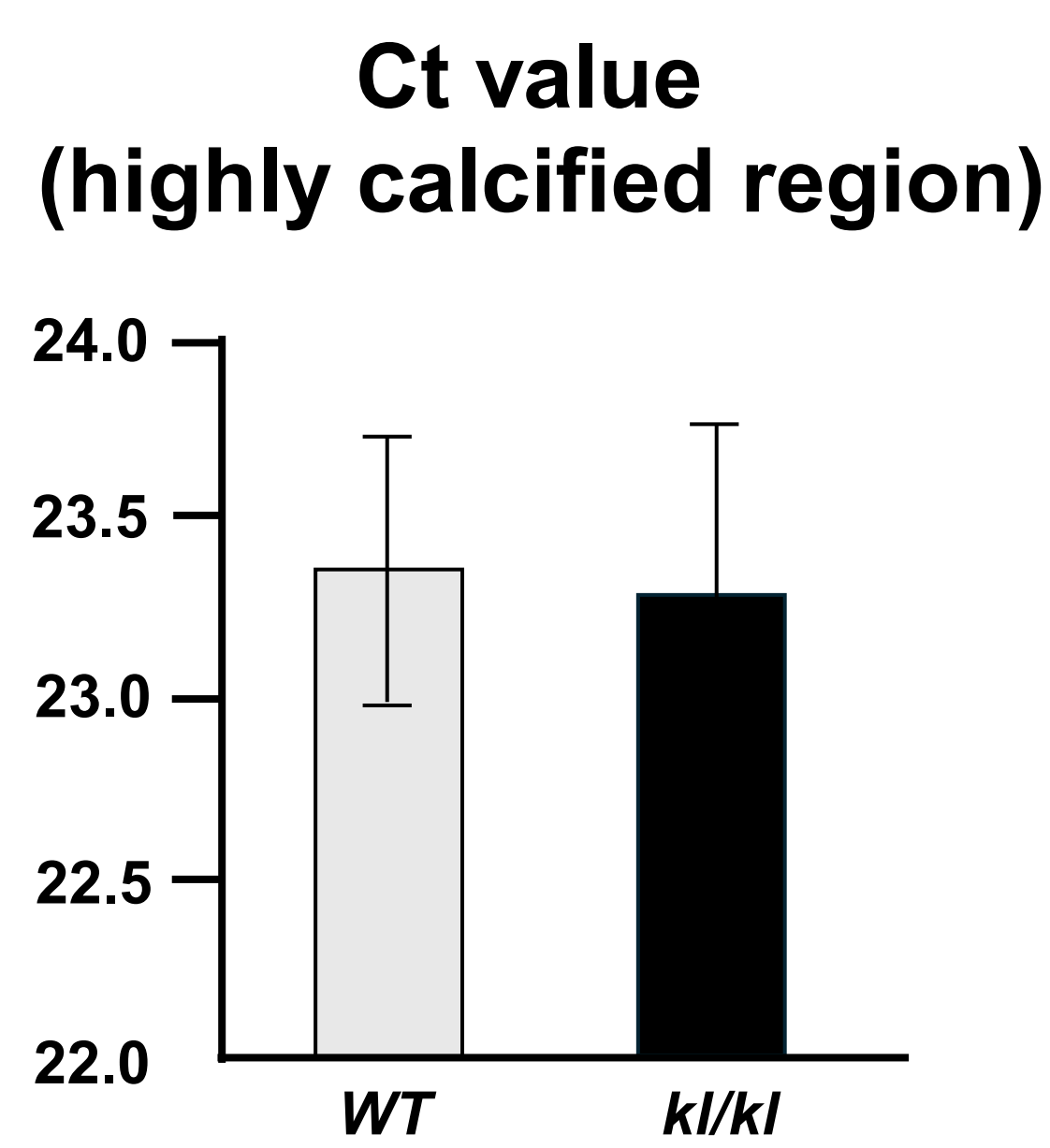

	Ct value	CV (%)
WT	23.35 ± 0.36	1.52
kl/kl	23.28 ± 0.46	1.99

Supplementary Figure 2

Target gene	Assay ID
Acan	Mm00545794_m1
Acta2	Mm00725412_s1
Alpl	Mm00475834_m1
Atf4	Mm00515324_m1
Bmp2	Mm01340178_m1
Bmp4	Mm00432087_m1
Casr	Mm00443375_m1
Col2a1	Mm01309565_m1
Col10a1	Mm00487041_m1
Ctsk	Mm00484039_m1
Ctss	Mm01255859_m1
Eln	Mm00514670_m1
Elane	Mm00469310_m1
Enpp1	Mm01193761_m1
Fgfr1	Mm00438930_m1
Galnt3	Mm00489348_m1
Gapdh	Mm99999915_g1
Lrp5	Mn01227476_m1
Lrp6	Mm00999795_m1
Mgp	Mm00485009_m1
Mmp2	Mm00439498_m1
Mmp9	Mm00442991_m1
Phospho1	Mm07301398_m1
Pth1r	Mm00441046_m1
Runx2	Mm00501584_m1
Slc20a1	Mm00489378_m1
Slc20a2	Mm00660203_m1
Sox9	Mm00448840_m1
Sp7	Mm04209856_m1
Spp1	Mm00436767_m1
Wnt1a	Mm01300555_g1
Wnt3a	Mm03053669_s1

Supplementary Table 2

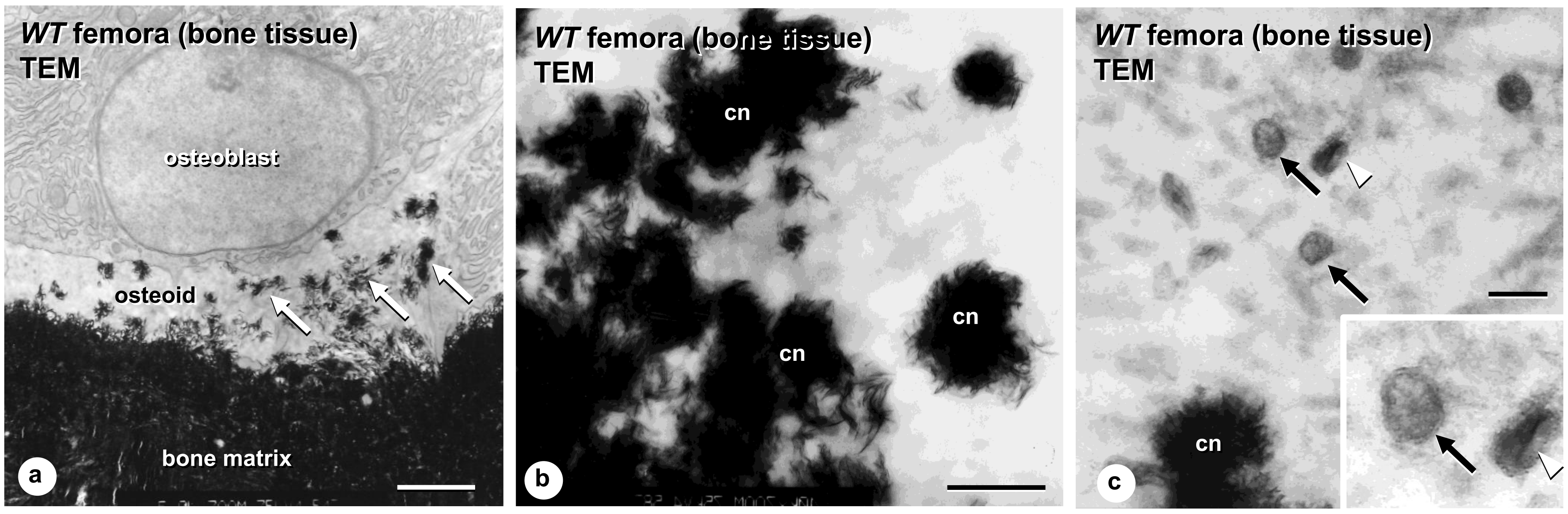

Supplementary Figure 3

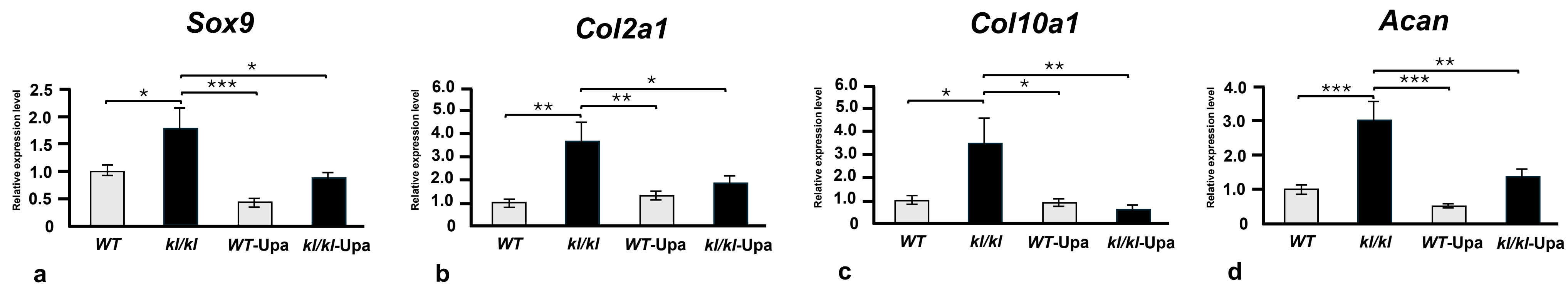

Supplementary Figure 4
